# Stem Cell Therapies for Central Nervous System Trauma: The 4 Ws—What, When, Where, and Why

**DOI:** 10.1093/stcltm/szab006

**Published:** 2022-02-19

**Authors:** Xiaofei Li, Erik Sundström

**Affiliations:** Department of Neurobiology, Care Sciences and Society, Karolinska Institutet, Stockholm, Sweden

**Keywords:** spinal cord injury, traumatic brain injury, neural stem cells, cell therapy

## Abstract

Traumatic injury of the central nervous system (CNS) is a worldwide health problem affecting millions of people. Trauma of the CNS, that is, traumatic brain injury (TBI) and spinal cord injury (SCI), lead to massive and progressive cell loss and axonal degeneration, usually with very limited regeneration. At present, there are no treatments to protect injured CNS tissue or to replace the lost tissue. Stem cells are a cell type that by definition can self-renew and give rise to multiple cell lineages. In recent years, therapies using stem and progenitor cells have shown promising effects in experimental CNS trauma, particularly in the acute-subacute stage, but also in chronic injuries. However, the therapeutic mechanisms by which transplanted cells achieve the structural and/or functional improvements are often not clear. Stem cell therapies for CNS trauma can be categorized into 2 main concepts, transplantation of exogenous neural stem cells and neural progenitor cells and recruitment of endogenous stem and progenitor cells. In this review, focusing on the advances during the last decade, we will discuss the major cell therapies, the pros and cons of these 2 concepts for TBI and SCI, and the treatment strategies we believe will be successful.

## Graphical Abstract

**Figure:**
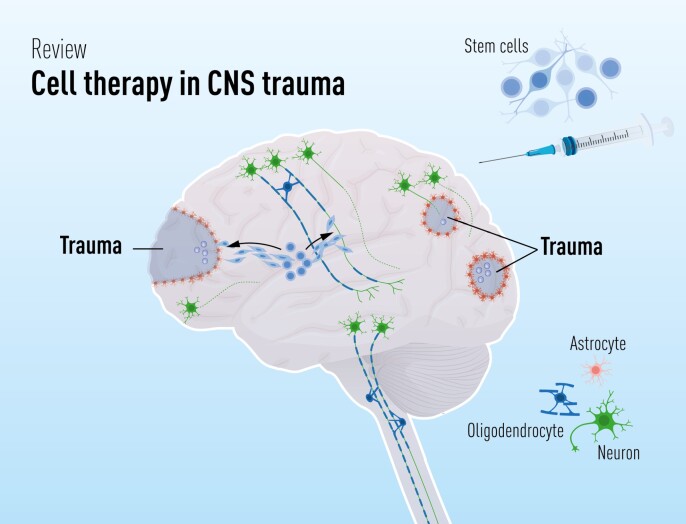
In this review, we summarize the pathology and pathophysiology of traumatic brain and spinal cord injuries. With this as the basis, we discuss major types of stem cell therapy for these insults, possible mechanisms, the role of endogenous stem cells, and what is known about timing and site of treatment. Finally, we discuss what future research should focus upon.

**Significance Statement**

Stem cell therapy provides a possibility to treat traumatic injuries of the brain and spinal cord that are not amenable to curative treatments today. Stem cells have the potential to reduce the initial injuries in the acute-subacute stages and replace lost tissue in chronic injuries. Different types of exogenous stem cells may be produced in cell culture and transplanted to select regions, and there are endogenous stem cells that respond to injury and could be used for treatment. In our review, we describe recent progress, and what we believe research should focus on.

## Introduction

Traumatic injuries of the brain and spinal cord affect millions of people all over the world, often with severe consequences for patients and families. According to the 2016 Global Disease Burden study the incidence 2016 was 27.1 (24.3-30.3) million for traumatic brain injury (TBI), and 0.93 (0.78-1.2) million for spinal cord injury (SCI), while the prevalence amounted to 55.5 (53.4-57.6) million and 27.0 (25.0-31.1) million for TBI and SCI, respectively.^1^ Hence, although TBI is much more common injury than traumatic SCI, the age-standardized prevalence of TBI is only twice that of SCI. Furthermore, since TBI has a higher mortality rate than SCI, the number of years “lived with disability” of patients with TBI is slightly smaller than SCI (8.1 vs. 9.5 million),^1^ suggesting that the long-term burden of SCI for patients, caregivers, and the healthcare systems may exceed that of TBI. Although very important advances in care and support have been made during the last decades, we still do not have any treatments that effectively prevent the injury process after trauma, or replace lost tissue. However, the development of cell therapy provides hope that this may change in a not-too-distant future. In this review, we will discuss how stem/progenitor cells can be used as therapy for TBI and SCI, and how the features of these 2 types of injuries will affect the strategies to develop treatments.

### Symptoms and Pathology of CNS Trauma

Severe central nervous system (CNS) trauma has major effects on several critical functions. Symptoms of TBI in each individual depend on the site of injury, but overall patients with severe TBI display a broad spectrum of motor, sensory, memory, cognitive, executive, emotional, psychiatric, and communication symptoms, including coma in severe cases. Patients with SCI experience complete or partial loss of motor and sensory functions below the injury level, and different degrees of bowel and bladder incontinence, sexual dysfunction, autonomic dysreflexia, spasticity, and neurogenic pain.^2,3^

TBI and SCI cause necrotic cell death early in the acute phase (0-2 days). The primary degeneration of neurons and glia is due to disruption of neural and vascular structures in the tissue by laceration, hemorrhage, ischemia, edema, and damage of the blood-brain barrier or blood-spinal cord barrier. During the acute phase, the first wave of inflammatory cells occurs, followed by the secondary degeneration during the subacute phase (2-14 days). The secondary degeneration is triggered by the release of cell constituents, acute inflammation, and ischemia. A cascade of degenerative processes such as the release of reactive oxygen species, glutamate toxicity, release of pro-apoptotic cytokines results in progressive degeneration.^4,5^ The loss of tissue due to the secondary degeneration often exceeds the primary degeneration. This is relevant to most treatment strategies since the affected tissue potentially can be salvaged if the degenerative cascade is prevented.

In the subacute phase, the glial scar mainly formed by reactive astrocytes seals off the core of the lesion with debris, myeloid cells, and fibroblasts,^6^ as well as inhibits axonal regrowth. Wallerian degeneration, an ordered process of axonal degeneration, sets in during the subacute phase but can persist for many months in human patients.^7,8^ Axon degeneration was recently shown to be mediated by specific molecular signaling,^9^ providing a potential target of neuroprotective treatments. Due to the slow infiltration of immune cells and the low capacity of the CNS to clear myelin, the accumulated myelin after injury leads to apoptosis of oligodendrocytes and further contributes to the failure of remyelination and regeneration.^4^

As the injury develops into the chronic phase, the glial scar is further organized, and a low-level degeneration of neurons and glia continues. While microglia take over the role of phagocytosis of cell debris from macrophages,^10^ chronic inflammation is established due to insufficient resolution, an inflammation that has widespread effects.^11^ Ongoing Wallerian degeneration results in continued deposition of cellular debris, maintaining inflammation and gliosis.^12^ The core of the lesion often develops into a degenerative cystic cavity. The processes that take place in the acute and subacute phases are often involved both in degeneration and in restoring the tissue homeostasis and enhancing regeneration. Consequently, they are difficult to target therapeutically. In the chronic phase, the milieu is, however, mainly detrimental, making it easier to develop therapies, including cell therapy, that does not interfere with endogenous repair mechanisms.

### The Location of CNS Trauma

**Figure 1.**
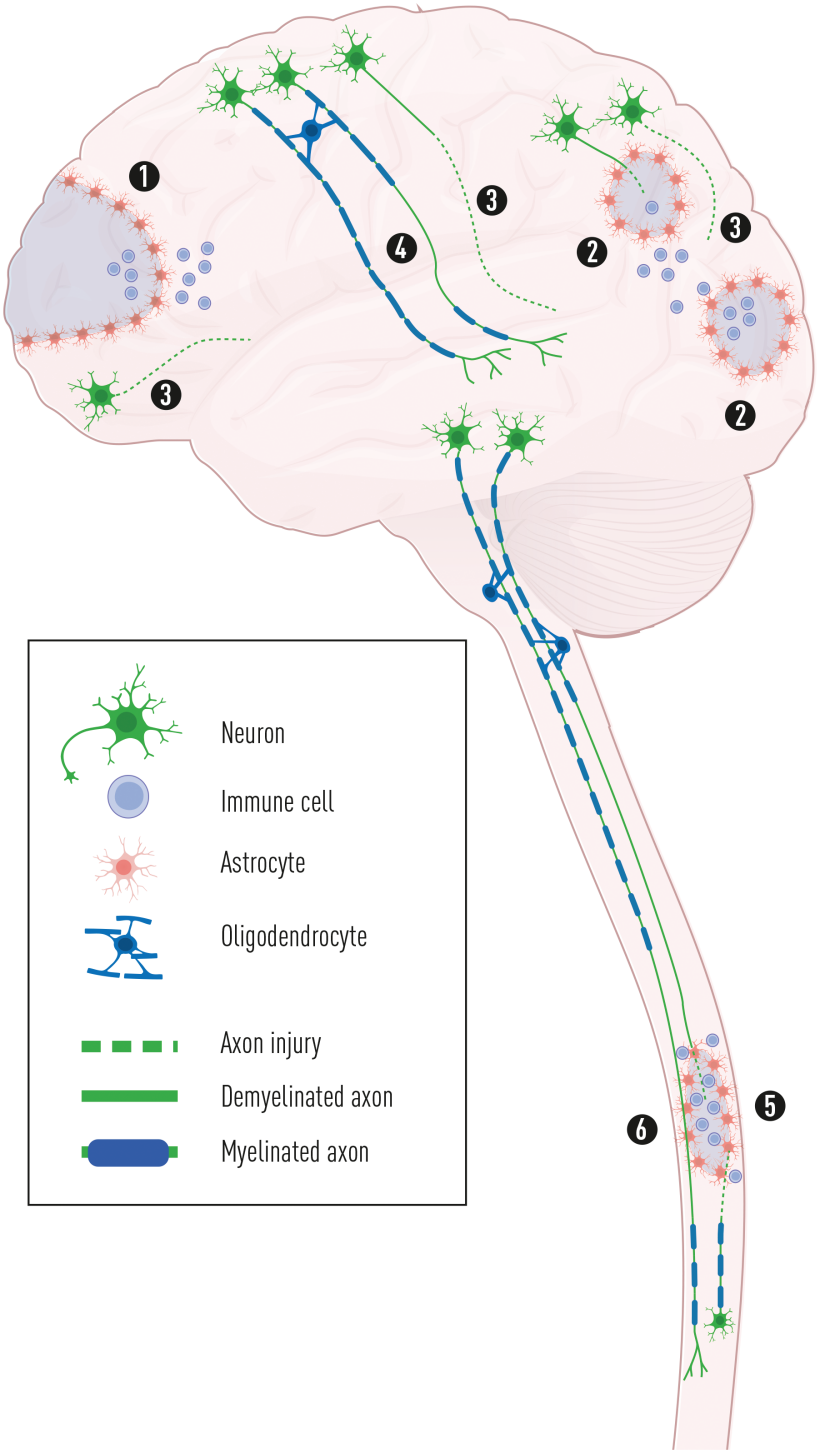
Schematic illustration of the pathology of TBI and SCI. In severe TBI, there are typically multiple regions of damage of the parenchyma, with the primary degeneration at the site of impact (1) as well as in other sites often opposite the impact (2). In addition to the pan-necrosis of cells due to the primary degeneration, the secondary degeneration takes place in compromised tissue close to the primary degeneration with axonal injury (3) and neuronal apoptosis and necrosis. In more distant white matter, DAI occurs with axonal degeneration (3) or demyelination (4). In contrast, even severe SCI is mainly restricted to one site with a central region of pan-necrosis and degeneration of ascending and descending axons passing through the region (5), with the secondary degeneration occurring in adjacent tissue. Axons affected by retrograde degeneration, and a large number of axons in the vicinity of the lesion core show widespread demyelination (6). Abbreviations: DAI, diffuse axonal injury; SCI, spinal cord injury; TBI, traumatic brain injury.

When strategies for cell therapy are discussed, the location of tissue lesions is critical. While SCI is primarily a focal injury, moderate to severe TBI is a multifocal injury, affecting the brain globally with several sites of degeneration (Fig. 1). Similar to SCI, there is a focal injury at the site of injury in TBI (“coup”), resulting in the primary and secondary degeneration. However, in TBI there are often more contusion sites with degeneration, often occurring opposite the primary contusion (“contre-coup”), as the brain bounces against the inner surface of the skull and deform. The impact of the skull leads to a pressure wave rapidly traversing the brain, which results in marked negative pressure at the contre-coup, and so-called contre cavitation,^13^ resulting in widespread degenerative changes.^14^ In addition, diffuse axonal injury (DAI), caused by detrimental acceleration/deceleration forces and shearing of neuronal and glial structures, leads to widespread degenerative changes in white matter,^15^ typically associated with extended periods of coma.^16^ Mortality rates after severe TBI are therefore relatively high, whereas patients with SCI typically survive. Importantly, loss of tissue in both TBI and SCI is the combined result of the primary and secondary degeneration.

### Potential Cell Therapy for CNS Trauma

CNS trauma represents currently incurable conditions, with low limited regenerative potential of the mammalian CNS. As stem cells have the potential of self-renewal and differentiation into multiple cell lineages, stem cell therapy has become one of the major strategies for developing treatments for CNS trauma. But what type of therapy has the best potential? In the field of stem cell therapy, there are 2 main strategies that are extensively studied: (1) stem/progenitor cell transplantation (exogenous) and (2) recruitment of resident stem and progenitor cells (endogenous). Since most of the studies focus on the acute/subacute phase, we will first discuss the pros and cons of different stem cell therapies carried out in this phase, then discuss therapies for chronic injuries. Due to the limited space, we can only highlight a fraction of all the important studies in this field. For this review, we performed searches in PubMed of publications in English from 2010 and later, including pre-prints of a few of the most recent studies. We included experimental and clinical studies as well as clinical trials.

## Cell Transplantation in CNS Trauma

**Figure 2.**
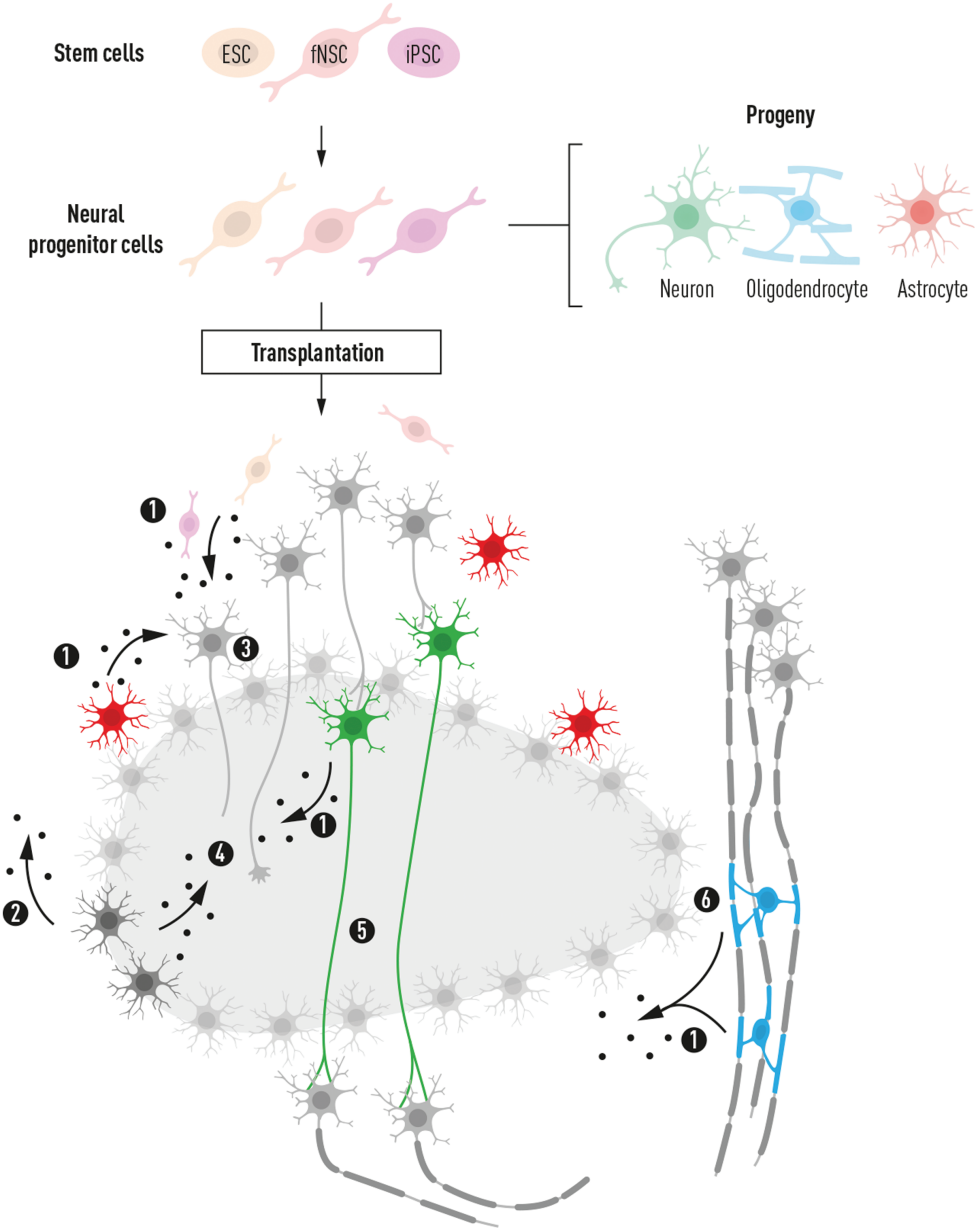
Illustration of potential effects of neural progenitor cells (NPCs), shared by NPCs derived from embryonic stem cells (ESCs), induced pluripotent cells (iPSCs), and from the embryonic-fetal brain. All NPCs derived from these sources have the capacity to differentiate into neurons, oligodendrocytes, and astrocytes. Common to all stem/progenitor cells studied is the expression, and probable/demonstrated release of various growth factors (1), which also occurs from endogenous astrocytes (2). The release of growth factors can support injured neurons (3) and enhance axonal regeneration (4). Neurons emanating from differentiating NPCs have the potential to bridge the injured area, relaying neuronal signaling across a region of degeneration (5), while NPC-derived oligodendrocytes can remyelinate demyelinated axons (6). All endogenous cells are illustrated in gray color while transplanted NPCs and their differentiated progeny are indicated in colors depending on the type of cells.

Cell transplantation has been extensively studied as a possible treatment in CNS trauma for more than 30 years.^17^ The underlying hypotheses are that transplanted cells (1) differentiate into functional neurons and glial cells, replace lost tissue and restore functional networks, or (2) provide factors to inhibit degeneration, repair the microenvironment of the injured CNS or enhance regeneration (Fig. 2). As we will discuss below, stem and progenitor cells can theoretically benefit the injured CNS through several mechanisms. Neural stem cells (NSCs) and neural progenitor cells (NPCs) have been shown to differentiate and replace lost cells.^18^ Release of growth factors that improve host cell survival after injury and/or enhance regeneration is a common feature of stem cells,^19^ as is the capacity to modulate immune mechanisms.^20,21^ To continue the development of more efficient cell therapy it is important to disentangle the different mechanisms of action, and determine which mechanism(s) that are important for therapeutic effects.

### Embryonic and Fetal Neural Stem and Progenitor Cells

Embryonic stem cells (ESCs) are obtained from early-stage embryos, and human ESCs are derived from discarded supernumerary embryos after in vitro fertilization.^22^ ESCs have unlimited potential to self-renew and can theoretically generate all stem and progenitor cells.^23^ Due to the uncertain fate of transplanted pluripotent cells, and almost inevitable tumor formation,^24^ ESCs are not transplanted in CNS trauma. Instead, NPCs restricted to neural lineages are derived from ESCs (hereafter termed eNPCs) using appropriate protocols for neural induction. NPCs can also be isolated from embryonic-fetal CNS tissue (hereafter termed fNPCs), including the subventricular zone (SVZ) and hippocampal subgranular zone (SGZ) in the brain, and the ventricular zone of the spinal cord.^25-27^ Human fNPCs are retrieved from clinical routine abortions.^18,28^

NPCs have the potency to differentiate into neurons and glia after transplantation to CNS injuries, and thereby replace lost tissue. Rodent studies of acute-subacute SCI showed that engrafted porcine eNPCs^29^ and human fNPCs^30-32^ can differentiate into neurons, integrate with host neural circuits, and contribute to functional recovery. Spinal cord-derived fNPCs differentiate more efficiently into neurons after transplantation than forebrain-derived fNPCs, indicating that the choice of regional NPCs is important for the recovery after CNS trauma.^33^ In a series of studies, the Tuszynski Lab showed that NPCs transplanted in a fibrin matrix supplemented with growth factors improve functional recovery after SCI in rodents and nonhuman primates. Importantly, the neurons derived from primate eNPCs^34^ and human fNPCs^35^ integrated structurally and functionally with the host neuronal circuitry through new synapses and served as relays to convey signals from rostral spinal levels across the lesion area.

Although the local environment changes considerably from the acute to chronic stage, which affects the conditions for successful regeneration,^36^ cell replacement can be effective also in chronic injuries. In a recent rodent study, transplantation of human directly reprogrammed NPCs derived from somatic cells and non-genetically biased toward oligodendrocyte differentiation, and combined with a treatment to facilitate axonal regeneration, improved functional outcome in a rat model of chronic SCI.^37^ With respect to clinical trials, human chronic patients with SCI were transplanted with an fNPC cell line (NSI-566), and the results showed that transplantation of fNPCs was feasible without post-surgery complications and serious adverse events. However, only 2 patients out of 6 displayed minor functional recovery.^38^ A prematurely terminated study sponsored by Stem Cells Inc. included 29 patients with cervical and thoracic SCI treated with intraspinal transplantation of fNPCs. While the treatment apparently was safe, no clear functional improvements were reported.^39^

As mentioned, it is crucial to identify the mechanisms of action. There is a cascade of biochemical and cellular processes in the acute-subacute stages that can be affected by transplanted NPCs with reduced degeneration as a result. Several transplantation studies of murine^40^ and human^41,42^ NPCs have shown improved functional recovery that can be attributed to neuroprotection, that is, reduced degeneration of compromised host neurons. Neuroprotection is a key mechanism by which fNPCs can improve functional outcomes after experimental SCI, and human fNPCs derived from the spinal cord are more effective than fNPCs from the forebrain.^33^ Several studies have specifically addressed the secretion of trophic factors as involved in neuroprotection,^40,43,44^ and release of neurotrophic factors from eNPCs and fNPCs has indeed been shown to modulate the microenvironment to reduce inflammation and increase neuronal survival in SCI.^45,46^ Still, additional mechanisms may be involved in the protection of host tissue such as homeostatic support of compromised host cells through gap junctions with transplanted stem/progenitor cells.^47^ Transplanted cells probably support neurons and glia by several mechanisms.

Similarly, in TBI do eNPCs and fNPCs show therapeutic effects through several mechanisms. Mouse fNPCs transplanted to a mouse TBI model reduced the number of microglia.^48^ In a more extensive study mouse fNPCs were acutely transplanted to rat TBI. fNPCs reduced neuronal apoptosis in the cerebral cortex and improved motor function, presumably by increasing Bcl-xL expression.^49^ Regarding inflammatory mechanisms, there has been an increasing focus on microglia in SCI and TBI in recent years, and the roles of the M1 (pro-inflammatory) and M2 (anti-inflammatory) phenotypes in degeneration and repair.^50,51^ Human NPCs have the ability to reduce inflammation after TBI in mice, specifically by increasing the M2/M1 ratio.^52^ Of particular relevance to clinical application is a recent study on freshly thawed cryobanked human eNPCs implanted in mice as late as 4 weeks after TBI. The transplanted cells increased host neuronal survival, reduced neuroinflammation, and improved cognitive functions.^53^

However, despite the efficacy of NPC transplantation in pre-clinical studies and the encouraging safety profile derived from the clinical trials, several concerns remain, such as tumor formation and ethical concerns. Regarding the former, to our knowledge there are no reports on tumor formation after transplantation of fNPCs to animals or in clinical trials of SCI cell therapy.^31,35,38,54^ eNPCs are associated with more obvious risks associated with remaining pluripotent cells.^55^ Studies using recent protocols suggest that eNPCs can be used for transplantation without obvious tumor formation.^29,34^ However, reliable evaluation of the tumor risk requires longer observation periods than is commonly used in transplantation experiments. It should also be recognized that suboptimal immune suppression in animal studies could lead to the rejection of pluripotent cells, thereby obscuring a tumor risk. Other concerns related to clinical use are that immunosuppression is required after heterologous transplantation therapy, and further complications could occur.^38,56^ In addition, fNPC lines are not immortal,^57^ suggesting that continuous tissue collection of aborted human fetuses is required if fNPC transplantations would become a standard treatment in the future. Since the use of fertilized oocytes and embryonic/fetal tissue for research and clinical regenerative treatments is controversial or prohibited in some countries, experimental research on eNPCs and fNPCs will not become the clinical practice in all countries.

### Induced Pluripotent Stem Cells (iPSCs)

Mouse and human somatic cells can be reprogrammed to iPSCs using reprogramming factors (Oct3/4, Sox2, Klf4, and c-Myc).^58,59^ As iPSCs can be obtained from the patient to be transplanted, iPSCs provide a therapeutic possibility with lower risks of immune rejection compared to hNPCs. In recent years, animal studies have provided compelling evidence that grafted iPSCs give rise to neurons and oligodendrocytes. iPSCs-derived neurons extend axons with synapses and can serve as relays between intact neurons on 2 sides of a lesion by integrating into neural networks, similar to fNPCs and eNPCs. Several studies have shown motor recovery after transplantation of mouse^32^ and human^32,60-64^ iPSCs, and a recent study on human iPSC-derived NPCs grafted to chronic SCI in mice showed significant functional improvement, albeit in combination with a drug to inhibit gamma-secretase.^65^ There are unfortunately far fewer studies on iPSC-derived cells transplanted after TBI. The studies have mainly demonstrated the feasibility of rat^66^ and human^67^ iPSC transplantation after TBI. One recent study on acute (1-day post-injury) transplantation of human iPSC-derived NPCs in a mouse contusion model found no positive functional effects.^68^

Similar to ESCs there are safety concerns with iPSCs, since any remaining pluripotent cells may lead to tumor formation.^69^ Recently, protocols for efficient differentiation of ESCs and iPSCs have been developed. In SCI transplantation studies, NPCs derived from iPSCs using modified reprogramming cocktails have shown that the tumor risk can be eliminated.^62,70^ Pre-treatment and selection of iPSC cultures showed better differentiation into functional oligodendrocytes.^71^ However, the functional recovery was still limited after the transplantation,^64^ and standardization is still a challenge due to the large number of protocols used. Another limitation of iPSCs for acute-subacute treatments is the duration of reprogramming and cell expansion. iPSC-derived NPCs from patient tissue can take months to produce, thereby limiting autologous transplantation to chronic stages. To overcome this limitation, the iPSC research institute at Kyoto University has created a cell bank of clinical-grade iPSCs from a large number of donors to provide clinical trials with cells that are human leukocyte antigen- (HLA-) matched to the majority of the Japanese population, thereby reducing the need for immunosuppressive treatments.^72^ Hypoimmunogenic cells with inactivated MHC genes and over-expressed CD47^73^ is another solution to provide cryobanked cells that can easily be used for clinical trials in SCI, TBI, and other disorders.

### Mesenchymal Stem Cells (MSCs)

The aim of MSC transplantation for SCI and TBI is usually to suppress inflammation, and take advantage of the secreted or membrane-bound factors that can provide neuroprotection and promote regeneration. MSCs can be isolated from different sources, including bone marrow, umbilical cord, amniotic fluid, and adipose tissue. Autologous MSCs can be used to avoid rejection and due to their immunomodulatory effects,^58^ even allogeneic MSCs have a low risk of rejection. There are no major ethical concerns using MSCs.^74^ MSC transplantation may reduce the secondary degeneration through the secretion of trophic factors including vascular endothelial growth factor (VEGF), nerve growth factor (NGF), glial cell-derived neurotrophic factor (GDNF), and brain-derived neurotrophic factor (BDNF).^75,76^ Animal studies have shown that MSCs can suppress inflammation and immune cell activity.^2^ For example, bone marrow MSCs exert anti-inflammatory effects, partly by enhancing a transition of M1 pro-inflammatory macrophages into M2 anti-inflammatory macrophages, to support regeneration.^77^ It has been suggested that the origin of MSCs is important for their effects after transplantation to the CNS. Some data suggest that umbilical cord-derived MSCs present the most immunomodulatory effects and potency for neuronal differentiation. It is, however, unclear if such differences translate to therapeutic differences. In a recent study on rat SCI, human MSCs derived from adipose tissue and the umbilical cord had similar therapeutic effects, and no cells differentiated into neurons.^78^ Evidence for neuronal differentiation of MSCs is limited to cell morphology and expression of markers. There are no reports on action potentials, a central neuronal feature, in cells originating from MSCs.

Although several clinical trials have shown that patients with SCI after MSC transplantations gain motor and sensory improvements,^79-83^ a phase III clinical trial showed that recovery was limited.^84^ Regarding TBI, human MSCs given intravenously in the acute phase after experimental TBI were neuroprotective, stimulated neurogenesis, and improved cognition by the release of Wnt3a.^85^ Considering the extended inflammation after CNS trauma, MSCs could theoretically be beneficial in chronic injuries. Experimental studies of intravenous MSC transplantation in chronic SCI have shown improved functional recovery.^86^ Recent interim results of a randomized double-blind study of bone marrow-derived MSCs implanted close to chronic TBI lesions indeed showed significant improvement in a motor impairment scale, but changes in a number of other functional scales were not significant.^87^

The plethora of MSC types resulting from the different sources and in vitro protocols used is a challenge for standardization of MSC transplantations. It is difficult to evaluate the efficacy, safety, and mechanisms due to the heterogeneity of MSCs, but the specific cell preparation used seems to be important for their therapeutic potency (see Kota et al^88^ for discussion).

Although the therapeutic profile of MSCs for both acute and chronic injuries is promising, more studies comparing different types of MSC in acute and chronic injuries are needed to understand the mechanisms of MSCs-based therapies for CNS trauma.

## Recruiting Endogenous Stem and Progenitor Cells in CNS Trauma

**Figure 3.**
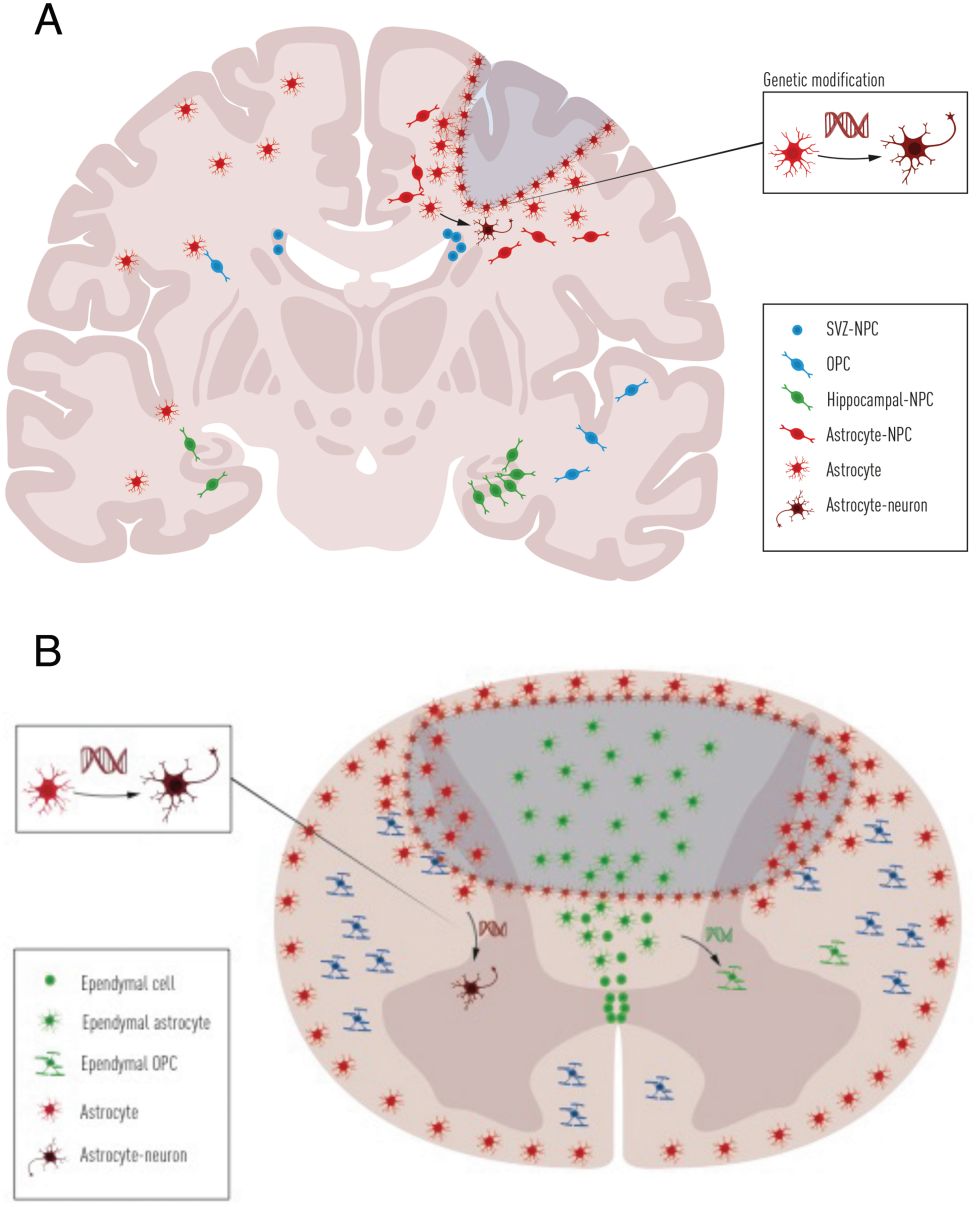
Illustration of the responses of endogenous neural stem cells and neural progenitor cells after neurotrauma. (A) After TBI, astrocytes, OPCs, and NPCs increase proliferation. Astrocytes near the lesion site form glial scar while some astrocytes in parenchyma can self-reprogram into neuroblasts. Upon gene modification, astrocytes can be reprogrammed into neurons. NPCs in SVZ might expand the stem cell pool slightly after TBI, while NPCs in the hippocampus largely increase their proliferation. (B) After SCI, ependymal cells, astrocytes, and OPCs all proliferate extensively. Ependymal cells around the central canal differentiate into astrocytes and a few oligodendrocytes, migrate to the lesion site, and constitute the core of the glial scar. Resident astrocytes form the border of glial scar at the lesion site, and OPCs differentiate into oligodendrocytes. If gene modification is introduced, more ependymal cells can differentiate into oligodendrocytes while resident astrocytes can convert into neurons. Abbreviations: NPCs, neural progenitor cells; OPCs, oligodendrocyte precursor cells; SVZ, subventricular zone; TBI, traumatic brain injury.

Although the transplantation studies conducted have provided some promising results for CNS trauma, concerns such as surgical complications, long-term immunosuppression, ethical issues, tumor formation still remain. In recent years, the discoveries of adult endogenous NSCs and NPCs, and their role in CNS regeneration have provided another strategy for therapy after CNS trauma. In adult mammalian CNS, cells derived from discrete regions of the brain and spinal cord can self-renew and differentiate into neurons and glia in vitro, suggesting that there are NSCs in the CNS beyond the developmental period (Fig. 3).^89,90^ Due to the lack of suitable tools, it was previously difficult to pinpoint what cell types have stem cell potential, and how different neural progenitors can promote recovery after SCI and TBI. Since 2005, different NSCs and NPCs can be genetically labeled in vivo using lineage tracing techniques based on Cre/loxP system to determine their origin and potential after CNS injuries.^91^

### Endogenous Neural Stem and Progenitor Cells in the Brain

In the adult brain, endogenous NSCs mainly reside in the SVZ and SGZ of the hippocampus. These NSCs contribute to adult neurogenesis under healthy conditions.^92^ After TBI, NSCs in SVZ can be activated for self-renewal in vitro^93^ but probably do not significantly contribute to the proliferative expansion of the SVZ,^94^ and mainly differentiate into glia instead of neurons.^94,95^ Several experimental studies have shown increased hippocampal neurogenesis after experimental TBI,^96,97^ which seems to be important for reactive plasticity in the hippocampus,^97^ and for the cognitive recovery after TBI.^98^ However, the migration of newborn neurons in the dentate gyrus has been reported to be aberrant^99^ and has been associated with the development of epileptic seizures.^100^ Thus, it is not clear how spontaneous neurogenesis contributes to functional recovery after TBI.

Besides NSCs, other progenitors were recently found to be activated and exhibit latent stem cell potential after TBI. Indeed, a subset of reactive astrocytes serve as a latent NSC population in the cortex, and show increased proliferation in vivo and in vitro after TBI, under the control of sonic hedgehog expression.^93^ Consequently, modulation of astrocytes or endogenous NSCs could serve as potential therapeutic approaches. For example, inhibiting Notch signaling in cortical astrocytes can induce reprogramming into neuroblasts,^101^ and induced expression of transcription factor NeuroD1 in astrocytes can directly reprogram them into neurons after TBI in vivo.^102^ Furthermore, by partly depleting microglia after TBI, repopulated microglia secrete several cytokines, which can activate endogenous hippocampal NSCs and promote neurogenesis.^103^

### Endogenous Neural Stem and Progenitor Cells in the Spinal Cord

In contrast to the brain, there is no clear evidence of neurogenesis in the normal or injured mammalian spinal cord. Using lineage-tracing mouse models, ependymal cells lining the central canal were found to self-renew and differentiate into neurons, astrocytes, and oligodendrocytes in vitro but undergo low proliferation in vivo under healthy conditions. However, after SCI ependymal cells proliferate extensively, differentiate into astrocytes and oligodendrocytes, but not neurons, and migrate to the lesion site to contribute to wound healing.^56,104^ Genetic manipulation of ependymal cells to block proliferation after SCI showed that ependymal cells are essential for spinal cord repair by contributing to glial scar formation and secretion of neurotrophic factors.^105^ In humans, ependymal cells also exhibit NSC features from young to mature adults.^106-108^ These studies suggest that ependymal cells can be targeted for regeneration regardless of the age of the patient with SCI.

Juvenile ependymal cells show higher stem cell potential after SCI compared to adults. Their contribution to SCI repair is regulated by the master transcription factor Forkhead Box J1 (FoxJ1).^27^ Thus, ependymal cells and FoxJ1 are potential therapeutic targets in SCI. Furthermore, overexpression of *olig2* in ependymal cells induces differentiation into oligodendrocytes in vivo to remyelinate axons and improve axon conduction after SCI.^109^ Interestingly, a recent study showed that repetitive magnetic transcranial stimulation can activate the stem cell potential of ependymal cells non-invasively, and promote functional recovery.^110^ Other glial cells may also be targets for regenerative therapies in SCI. For example, modulating endogenous astrocytes and the glial scar could enhance axonal growth and functional recovery after SCI.^111^ In addition, expressing *Sox2* or *Zfp521* in astrocytes results in lineage conversion from astrocytes to neurons in vivo,^112^ or reprogramming into neural progenitors, with functional improvements in experimental SCI.^113^

## Time and Place

Two of the critical questions in cell therapy are “when?” and “where?” When to treat relates to the therapeutic time window which depends on the dominant treatment effect of the therapy discussed. Most cell transplantation studies have been performed in the acute or subacute phase of TBI or SCI. However, there are many reasons to focus more on chronic treatments. Patients in the acute/subacute phase may not be medically stable to allow invasive treatments, and may not have the cognitive capacity or emotional status to decide on a potentially hazardous treatment. Patients improve spontaneously during the first 6-12 months, acute intervention studies must therefore include a large number of patients to detect significant effects, preferably with sham-treated controls. For acute and subacute treatments there is not sufficient time for in vitro expansion of iPSC-derived NPCs from the patient. For studies in the chronic phase with patients that have more stable symptoms, the treatment groups can be much smaller with similar statistical power. The logistics of a chronic study are also considerably less complicated.

However, the chronic situation apparently is biologically challenging, and treatment efforts usually fail. While an experimental study reported that transplantation of human fNPCs in acute, subacute, and chronic SCI all resulted in significant functional improvement,^114^ we have found that the functional and structural improvement by human fNPCs in SCI rats was lost when transplantation was delayed by 7 weeks.^41^ Other studies have shown that rodent NPCs transplanted in chronic SCI fail to improve motor function.^43,115^ The lack of effect of chronic NPC transplantation is probably due to host-specific factors since RNA sequencing analysis showed that NPCs transplanted to chronic SCI animals retain high neural differentiation capacity.^43^ Successful stem cell therapy in the chronic phase probably requires modulating the microenvironment to support graft survival and integration, as well as maintaining regenerative axonal growth and synaptogenesis. Regarding endogenous NSCs and NPCs, they have also mainly been investigated in the subacute phase, as most of the endogenous stem cells in the CNS are activated and proliferate during 2 weeks after injury.^56,93^ More studies on endogenous stem/progenitor cells should be done in the chronic stage.

The question “where?” is difficult to answer. Almost no studies address this issue, and the place of cell transplantation varies considerably from study to study. In a few studies, cells were administered systemically, but in experimental studies, NPCs have usually been injected into the parenchyma. Unfortunately, in many studies, the cells were injected at several sites without any comparison of the therapeutic effects of different transplantation sites. We could only find one proper comparison of implantation sites, addressing intrathecal and intraspinal injections of human iPSC-derived NPCs in rat SCI.^116^ Loss of tissue was greater after intraspinal implantation but functional recovery was better.

This problem is unfortunately also a concern in many clinical trials. For example, in the published clinical trials on MSC in SCI, a different number of injections have been used, with injections in and/or above the lesion area, combinations of intraspinal and intrathecal cell injections, or even intraspinal combined with intravenous, making it very difficult to draw mechanistic conclusions related to the site of implantation.^79-83^ Although it is possible that the migration of engrafted cells makes the actual site of injection less critical, for the sake of clinical translation it would be advantageous if standardized injection protocols could be used, and studies comparing different injection protocols is clearly needed.

## Conclusion

After decades of research, CNS trauma is still incurable. However, a wealth of published research shows that cell therapy has the potential to change this situation, thus the answer to the question “Why?” We continue to see important progress leading to deeper understanding of processes involved. In acute-subacute rodent studies, transplantation of exogenous stem cells or modulation of endogenous stem and progenitor cells have been shown to protect tissue at risk, compensate for cell loss, repair neural circuitry, and promote functional recovery. Still, the importance of the different mechanisms is not sufficiently understood. We believe available research data show that most of the effects seen in acute-subacute transplantation paradigms are due to the release of various factors, and changes of the microenvironment in favor of cell differentiation. Similar effects could possibly be achieved using small molecules or biologicals considering the advances in molecular design during recent years.

In chronic injuries, however, tissue is already lost, and can only be replaced by exogenous or endogenous stem cells, probably as multimodal therapies, combining treatments to enhance graft survival, appropriate fate choices, differentiation, and functional integration. We therefore suggest more efforts should be put into developing cell therapy for chronic TBI and SCI. While TBI is more complex with multifocal injuries and a multitude of degenerative changes, results from SCI research will also be important for better understanding of common mechanisms.

Finally, more clinical studies should be performed based on the most promising experiments, and replicated by independent groups. This is of course more easily said than done. Clinical trials are always complicated and very expensive, although the cost for chronic studies is lower. Funding for academic trials is rarely available and commercial actors may find that markets other than CNS injuries are more attractive with less risks. Addressing these and related issues is a task the scientific community will have to put a stronger focus on in the future.

## Data Availability

No new data were generated or analyzed in support of this research.
